# Major Basic Protein Deposition Without Eosinophilic Infiltration in Hypercontractile Esophagus: A Case Report

**DOI:** 10.1002/deo2.70206

**Published:** 2025-09-29

**Authors:** Tetsuya Tatsuta, Keinosuke Hizuka, Shigeharu Ueki, Masatoshi Kaizuka, Shinji Oota, Keisuke Hasui, Hidezumi Kikuchi, Hiroto Hiraga, Daisuke Chinda, Hirotake Sakuraba

**Affiliations:** ^1^ Department of Gastroenterology Hematology and Clinical Immunology Hirosaki University Graduate School of Medicine Aomori Japan; ^2^ Department of General Internal Medicine and Clinical Laboratory Medicine Akita University Graduate School of Medicine Akita Japan; ^3^ Division of Endoscopy Hirosaki University Hospital Aomori Japan

**Keywords:** eosinophilic inflammation, hypercontractile esophagus, major basic protein, motility disorder, peroral endoscopic myotomy

## Abstract

Hypercontractile esophagus is a motility disorder characterized by excessive contractions in the esophageal body. Certain cases of hypercontractile esophagus exhibit eosinophilic infiltration in the muscle layer; however, its clinical significance is unclear. Here, we report a case of hypercontractile esophagus with possible eosinophilic inflammation despite the absence of eosinophilic infiltration on hematoxylin and eosin staining. A 75‐year‐old man presented with dysphagia, primarily triggered by the ingestion of meat. Esophagogastroduodenoscopy showed abnormal peristalsis of the esophageal body, while the lower esophageal sphincter function remained normal. High‐resolution manometry confirmed hypercontractile esophagus, according to the Chicago Classification version 4.0. As symptoms persisted despite medical treatment, the patient underwent peroral endoscopic myotomy. Biopsies obtained from the inner circular muscle layer revealed no notable eosinophilic infiltration on hematoxylin and eosin staining. However, immunofluorescence staining for major basic protein (MBP), a cytotoxic eosinophil granule protein that persists in tissues, showed patchy depositions. Corresponding counterstaining revealed collapsed nuclei surrounded by eosinophilic material, suggesting MBP release via eosinophil cytolysis. This case demonstrated that immunostaining for eosinophil granule proteins may uncover eosinophilic activity in the esophageal muscle layer, even in the absence of eosinophils. While the precise pathogenic role of eosinophilic inflammation in hypercontractile esophagus remains unclear, MBP deposition could reflect a localized immune‐mediated process contributing to motility disturbance. Further investigation is needed to determine the prevalence, mechanisms, and clinical implications of these findings in esophageal motor disorders.

## 1 Introduction

Hypercontractile esophagus is an esophageal motility disorder characterized by excessive contractions in the esophageal body [1]. According to the Chicago Classification version 4.0, it is diagnosed when ≥ 20% of swallows exhibit a distal contractile integral (DCI) > 10,000 mmHg·s·cm with normal integrated relaxation pressure (IRP) [2, 3]. Peroral endoscopic myotomy (POEM) is a viable treatment option for hypercontractile esophagus, particularly when medication fails, resulting in favorable outcomes [1].

Sato et al. reported eosinophilic esophageal myositis (EoEM) in patients with hypercontractile esophagus who underwent muscle layer biopsy during POEM. EoEM is characterized by eosinophilic infiltration of the esophageal muscular layer but not the mucosa [4]. However, not all cases of hypercontractile esophagus exhibit eosinophilic infiltration in the muscle layer, and its clinical significance remains unclear.

Here, we report a case of hypercontractile esophagus with marked major basic protein (MBP) deposition on muscle layer biopsy, despite the absence of eosinophilic infiltration on hematoxylin and eosin (HE) staining.

## 2 Case Report

**FIGURE 1:**
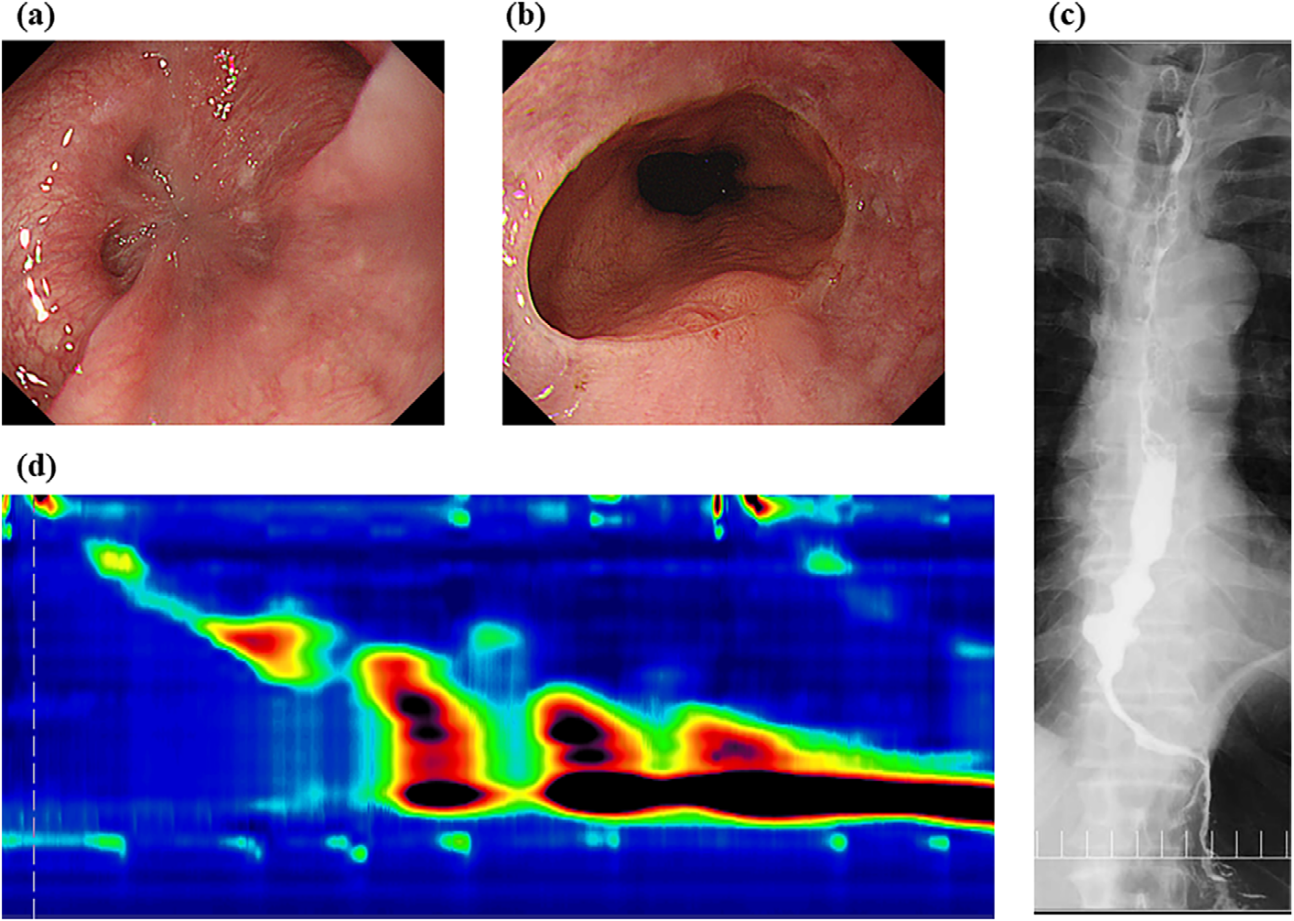
a: Esophagogastroduodenoscopy shows strong contractions in the middle to lower esophagus. b: Esophagogastroduodenoscopy shows no obvious stricture at the esophagogastric junction. c: Esophagography shows anterograde esophageal contractions and barium retention in the lower esophagus. d: High‐resolution manometry shows a distal contractile integral maximum of 12,243.9 mmHg/s/cm, >10,000 in three swallows, confirming the diagnosis of hypercontractile esophagus.

A 75‐year‐old man was referred for the evaluation and treatment of dysphagia following meat ingestion. His Eckardt score [5] was 7 (body weight loss, 1; dysphagia, 3; chest pain, 0; regurgitation, 3). The patient had a medical history of prostatic hypertrophy, hypertension, and cutaneous pruritus. His medications included nifedipine, tamsulosin, fexofenadine, and topical clobetasol propionate. He had no history of food or drug allergies. Laboratory tests revealed normal eosinophil counts (170.1/mm) and IgE levels (112.0 IU/mL). Esophagogastroduodenoscopy (EGD) revealed abnormal peristalsis in the esophageal body (Figure 1a), while no obvious stricture was observed at the esophagogastric junction (Figure 1b). Esophageal mucosal biopsies were obtained from three sites 4 weeks prior to POEM, and immunostaining for MBP was performed. However, neither intact eosinophils nor MBP deposition was identified.

**FIGURE 2:**
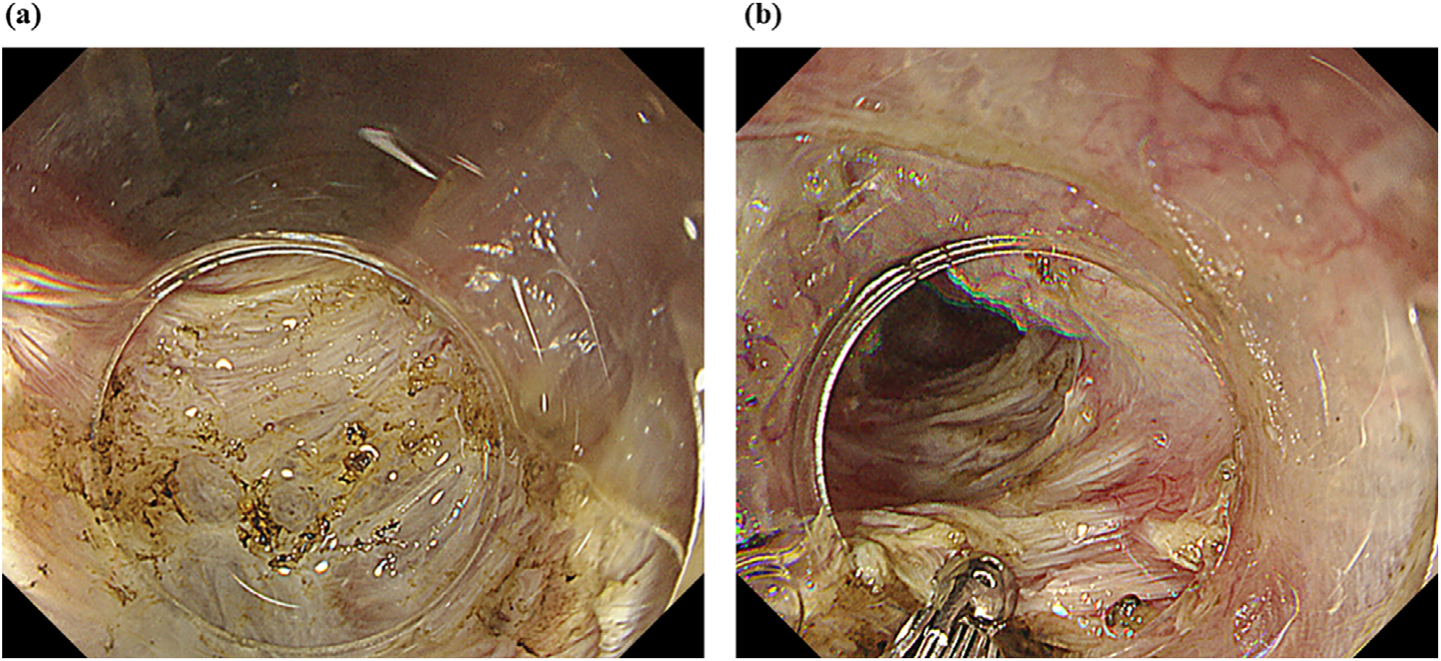
a: Peroral endoscopic myotomy (POEM) and incision of the inner circular muscle layer of the esophagus. b: Biopsies from the inner circular muscle layer of the esophagus during POEM.

Esophagography revealed antegrade esophageal contraction with barium retention at the same site (Figure 1c). High‐resolution manometry (HRM) showed findings consistent with a hypercontractile esophagus, as defined by the Chicago Classification version 4.0 [2]. The IRP was 18.1 mmHg, and the distal latency was 8.1 s, which were within normal ranges. The maximum DCI was 12,243.9 mmHg·s·cm, exceeding 10,000 mmHg·s·cm in 3 out of 10 swallows (Figure 1d). As the patient's symptoms persisted, POEM was performed (Figure 2a). Additionally, three biopsies were obtained from the inner circular muscle layer (Figure 2b) near the entry site of the POEM procedure, and three additional samples were obtained from more distal areas with strong muscular contractions.

**FIGURE 3:**
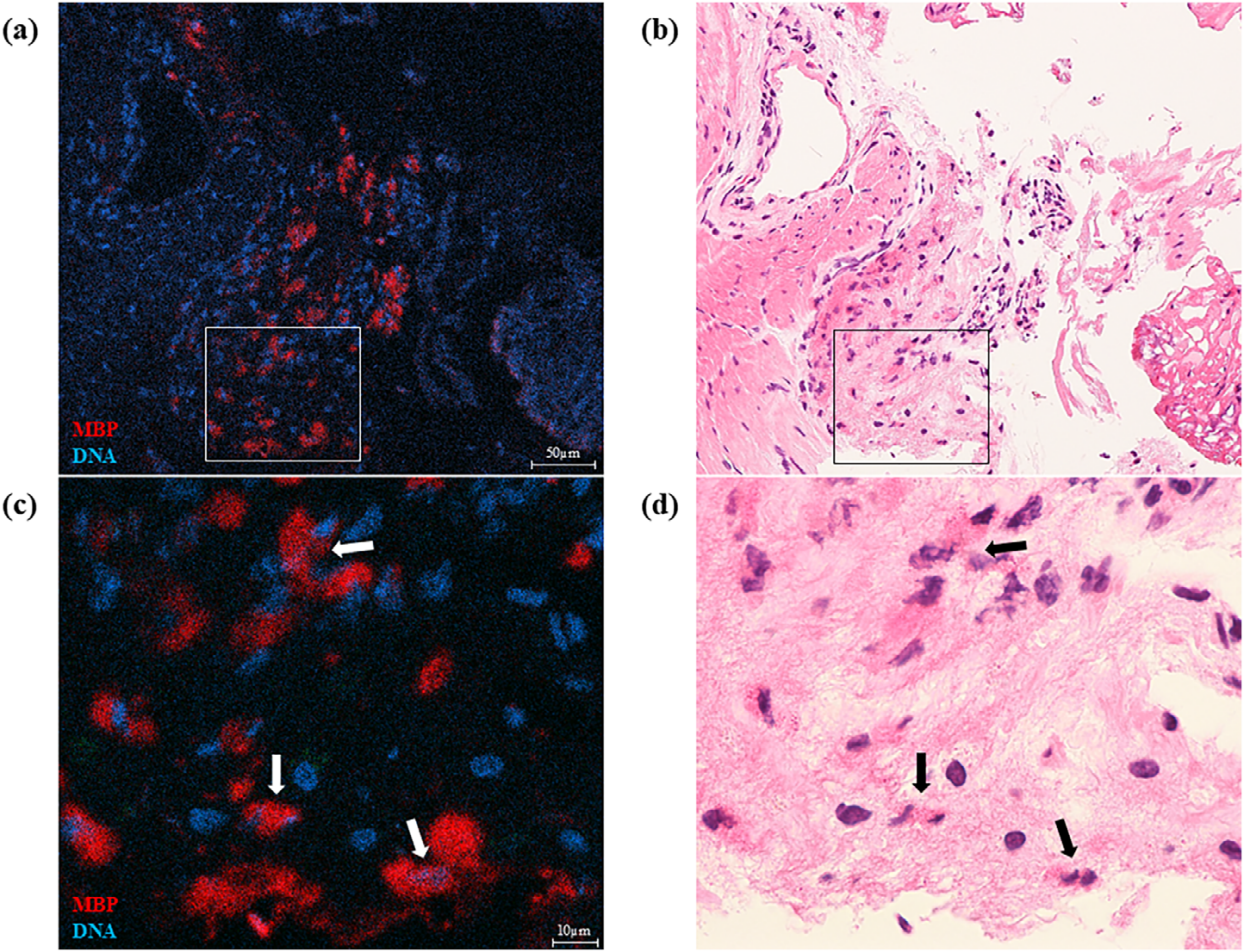
Histological analysis of the inner circular muscle layer of the esophagus. The boxed areas in panels (a, b) (×200) are magnified in panels (c, d) (×1000). Identical sections stained with anti‐major basic protein (MBP) antibody (red) and Hoechst 33342 (blue) (a, c), followed by hematoxylin and eosin staining (b, d). a, c: Patchy MBP deposition is evident. b, d: Hematoxylin and eosin staining reveals no intact eosinophils. The areas corresponding to the MBP deposition show collapsed nuclei surrounded by eosinophilic regions (arrows).

Initial HE staining showed no notable eosinophilic infiltration. To investigate the potential involvement of eosinophilic inflammation, immunostaining for MBP was performed. It revealed a patchy MBP deposition in the inner circular muscle layer (Figure 3a, c). Corresponding HE counterstaining showed collapsed nuclei surrounded by eosinophilic areas (Figure 3b, d), indicating tissue MBP deposition from cytolytic eosinophils.

**FIGURE 4:**
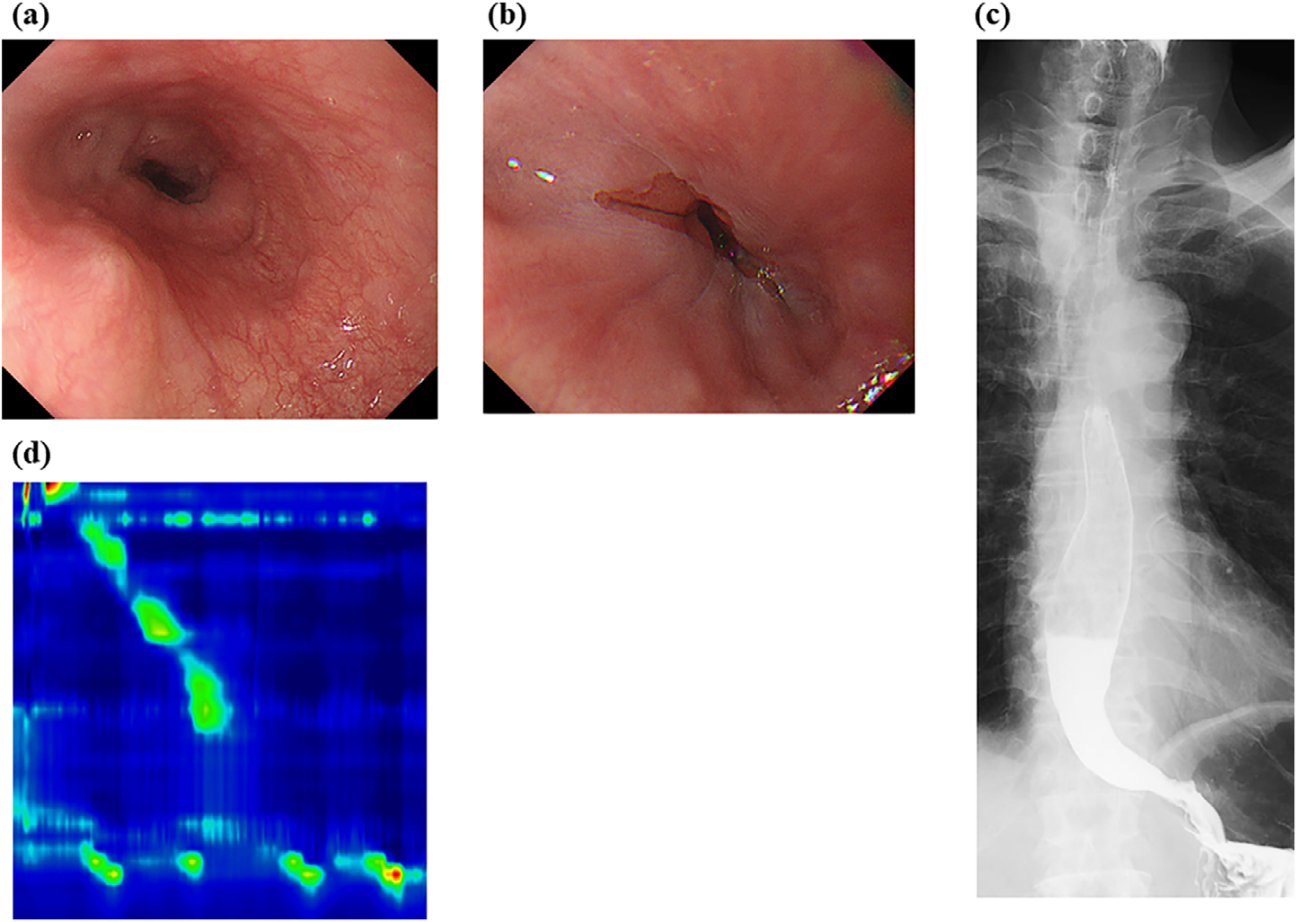
a: Esophagogastroduodenoscopy 2 months after peroral endoscopic myotomy (POEM) reveals no strong peristaltic contractions in the middle to lower esophagus. b: Esophagogastroduodenoscopy reveals no mucosal break in the esophagus. c: Esophagography 2 months after POEM shows improved barium transit through the esophagus. d: High‐resolution manometry 2 months after POEM shows no strong peristaltic contractions in the lower esophagus.

Two months after POEM, the patient's Eckardt score improved to 1 (body weight loss, 0; dysphagia, 0; chest pain, 0; regurgitation, 1), with no gastroesophageal reflux disease symptoms and no esophageal mucosal break on EGD (Figure 4). HRM showed resolution of the lower esophageal tonic contraction. One year later, the patient remained symptom‐free and experienced favorable outcomes.

## 3 Discussion

In this case, we observed MBP deposition in the inner circular muscle layer of the esophagus, despite the absence of eosinophilic infiltration on conventional HE staining. Eosinophils contain four major cationic proteins, including the MBP, which play a key role in eosinophil‐mediated inflammation. They release these granule proteins through various mechanisms, including piecemeal degranulation, exocytosis, and cytolysis [5]. Among these, cytolysis leads to extracellular deposition of granule proteins without retaining cellular morphology [6], making eosinophils potentially unrecognizable by HE staining. However, MBP remains detectable in tissues for several weeks using immunostaining [7]. Therefore, in our case, immunostaining revealed patchy MBP deposition in the muscle layer, with collapsed nuclei surrounded by eosinophilic granule proteins, consistent with eosinophilic cytolysis.

Immunostaining for eosinophilic granule proteins may reveal prior eosinophilic activity even in the absence of intact eosinophils, particularly when cytolytic eosinophils are predominant. Tissue deposition of MBP in the absence of eosinophil accumulation has been documented in multiple eosinophil‐associated conditions, including eosinophilic esophagitis (EoE) and hypereosinophilic syndrome with gastrointestinal involvement [8]. In EoE, the extent of MBP deposition correlates more strongly with clinical symptoms and tissue dysfunction than with eosinophil counts alone [9], underscoring its potential utility as a marker of disease activity. Although eosinophilic infiltration of the esophageal muscle has been reported in certain cases of hypercontractile esophagus [4], MBP deposition in this context has not been described. Therefore, our findings suggest that subtle eosinophil‐related immune processes—possibly involving cytolytic eosinophils—may contribute to tissue changes in certain patients with hypercontractile esophagus.

The pathophysiology of hypercontractile esophagus is multifactorial and incompletely understood. Proposed mechanisms include smooth muscle hyperreactivity, neural dysregulation, and mechanical or functional obstruction [10]. Identifying MBP deposition in this patient introduced eosinophil‐mediated tissue modulation as a possible additional contributor to these mechanisms.

The patient experienced significant symptom improvement following POEM alone, without anti‐inflammatory therapy. POEM is also effective in patients with EoEM [4], suggesting that MBP deposition may reflect a past immune event rather than an ongoing inflammatory process. Therefore, in some patients, eosinophil‐associated tissue changes may persist after the active immune phase has resolved, and esophageal dysmotility may become functionally independent of ongoing inflammation.

A limitation of this study was the lack of comparison with MBP‐stained muscle layer samples from healthy individuals. However, obtaining these biopsies is not ethically feasible during routine diagnostic endoscopy. They can only be obtained during invasive procedures such as POEM, which are not performed in healthy individuals. Furthermore, although key eosinophil‐associated mediators such as interleukin‐5, interleukin‐13, eotaxin‐3, and eosinophil peroxidase were relevant to this context, they were not assessed.

This case highlighted the potential for eosinophil granule protein immunostaining to reveal subtle, previously undetected inflammatory activity in the esophageal muscle of patients with hypercontractile esophagus. While the clinical implications of such findings remain uncertain, their identification may support further stratification of disease subtypes and inform future investigations into individualized management strategies.(Supporting Information)

## Author Contributions

T.T, M.K, S.O, K.H, H.K, D.C, H.H: Investigation. T.T, K.H: Writing–Original Draft Preparation. S.U, H.S: Writing–Review & Editing.

## Conflicts of Interest

The authors declare no conflicts of interest.

## Supporting information




**Supporting File**: deo270206‐sup‐0001‐SuppMat.pdf

## References

[1] D. Chinda , T. Shimoyama , S. Fujiwara , et al., “Assessment of the Physical Invasiveness of Peroral Endoscopic Myotomy During the Perioperative Period Based on Changes in Energy Metabolism,” Metabolites 13 (2023): 969. 37755250 10.3390/metabo13090969 PMC10536107

[2] R. Yadlapati , P. J. Kahrilas , M. R. Fox , et al., “Esophageal Motility Disorders on High‐resolution Manometry: Chicago Classification Version 4.0©,” Neurogastroenterology and Motility 33 (2021): e14058. 33373111 10.1111/nmo.14058 PMC8034247

[3] S. Kuribayashi , K. Iwakiri , T. Shinozaki , et al., “Clinical Impact of Different Cut‐off Values in High‐resolution Manometry Systems on Diagnosing Esophageal Motility Disorders,” Gastroenterol 54 (2019): 1078–1082. 10.1007/s00535-019-01608-3 31388756

[4] H. Sato , M. Takeuchi , K. Takahashi , et al., “Nutcracker and Jackhammer Esophagus Treatment: A Three‐case Survey, Including Two Novel Cases of Eosinophilic Infiltration Into the Muscularis Propria,” Endoscopy 47 (2015): 855–857. 25961439 10.1055/s-0034-1391985

[5] A. J. Eckardt and V. F. Eckardt , “Treatment and Surveillance Strategies in achalasia: An Update,” Nature reviews Gastroenterology & hepatology 8 (2011): 311–319. 21522116 10.1038/nrgastro.2011.68

[6] S. Ueki , R. C. N. Melo , I. Ghiran , L. A. Spencer , A. M. Dvorak , and P. F. Weller , “Eosinophil Extracellular DNA Trap Cell Death Mediates Lytic Release of Free Secretion‐competent Eosinophil Granules in Humans,” Blood 121 (2013): 2074–2083. 23303825 10.1182/blood-2012-05-432088 PMC3596967

[7] M. D. P. Davis , D. A. Plager , T. J. George , E. A. Weiss , G. J. Gleich , and K. M. Leiferman , “Interactions of Eosinophil Granule Proteins With Skin: Limits of Detection, Persistence, and Vasopermeabilization,” Journal of Allergy and Clinical Immunology 112 (2003): 988–994. 14610493 10.1016/j.jaci.2003.08.028

[8] S.‐I. Hagiwara , S. Ueki , K. Watanabe , K. Hizuka , and Y. Etani , “Case of Hypereosinophilic Syndrome With Gastrointestinal Involvement Showing Tissue Eosinophil Cytolysis,” Asia Pac Allergy 12 (2022): e37. 36452011 10.5415/apallergy.2022.12.e37 PMC9669462

[9] K. A. Peterson , G. J. Gleich , N. S. Limaye , et al., “Eosinophil Granule Major Basic Protein 1 Deposition in Eosinophilic Esophagitis Correlates With Symptoms Independent of Eosinophil Counts,” Diseases of the Esophagus 32 (2019): doz055. 31310661 10.1093/dote/doz055

[10] D. A. Patel , R. Yadlapati , and M. F. Vaezi , “Esophageal Motility Disorders: Current Approach to Diagnostics and Therapeutics,” Gastroenterology 162 (2022): 1617–1634. 35227779 10.1053/j.gastro.2021.12.289 PMC9405585

